# Antipyretics in the emergency department - intravenous paracetamol versus intramuscular diclofenac: a comparative study

**DOI:** 10.1186/cc13269

**Published:** 2014-03-17

**Authors:** N Purayil, N Vamanjore, F Paramba, O Mohammad, P Chandra

**Affiliations:** 1 Hamad Medical Corporation, Doha, Qatar

## Introduction

Fever is a common problem in adults visiting the emergency department (ED) [1]. Although it is important to treat the cause of fever, symptomatic management of fever is also necessary. Multiple studies are available on the efficacy of various antipyretics in children, but very little has been done in adults [1],[2]. Hence we aimed to compare the antipyretic efficacy of intravenous (i.v.) paracetamol and intramuscular (i.m.) diclofenac in adult patients presenting with fever to the ED.

## Methods

In this parallel-group, open-label trial, participants aged 14 to 75 years who had a temperature more than 38.5°C were enrolled and treated in the ED, Alkhor, Hamad Medical Corporation, during the period June 2008 to December 2011. Patients were randomly assigned to receive either 1,000 mg i.v. paracetamol or 75 mg i.m. diclofenac. The primary outcome was degree of reduction in mean oral temperature at 90 minutes and the secondary outcomes were degree of reduction at 30, 60 and 120 minutes. The efficacy of drugs was assessed by a superiority comparison. Analysis was done using intention-to-treat (ITT) principles.

## Results

In total, 139 patients received i.v. paracetamol and 150 received i.m. diclofenac. The mean age was 35.5 ± 14.24 and 36.4 ± 14.98 years in the i.m. diclofenac and i.v. paracetamol groups respectively. The majority of the patients were males in both groups. After 90 minutes both groups showed a significant reduction in mean temperature, i.m. diclofenac showing a greater reduction (-1.44 ± 0.43(95% CI -1.4, -2.5)) than i.v. paracetamol (-1.35 ± 0.46 (95% CI -1.3, -3.1) *P *< 0.0001). After 120 minutes, a significant difference was observed in the mean change from the baseline temperature between the groups, -1.81 ± 0.46 (95% CI -1.7, -2.9) in i.m. diclofenac versus -1.63 ± 0.55 (95% CI -1.5, -4.1) in the i.v. paracetamol group (*P *< 0.0001). Significant changes in temperature were observed in favor of i.m. diclofenac over i.v. paracetamol at each time point from 60 minutes through 120 minutes inclusive.

## Conclusion

Both i.m. diclofenac and i.v. paracetamol showed significant antipyretic activity, with diclofenac having a greater effect. In view of ease of administration, i.m. diclofenac can be used as a first-choice antipyretic in febrile adults in the ED.
